# 

**Published:** 1882-01

**Authors:** 


### CONTENTS:

PAGE.

ORIGINAL COMMUNICATIONS:

The Permanent Cure of Hernia. By W. H. Heath, M. D., . . . . . . 241
Perityphlitis or Perinephritis. Reported by Judson B. Andrews, M. D., . . . . 247

CLINICAL REPORTS:

Clinics at the Buffalo General Hospital by Professor Rochester. Reported by Dr. C. C. Fredericks, . . . . . . 253
A Case of Gouty Heart, with Notes of Autopsy. Reported by H. R. Hopkins, M. D., . . . . . . . . . 255
Treatment of Herpes Zoster. Reported by John Boardman, M. D., . . . . 257

TRANSLATIONS:

Contribution to the Causistry of Hereditary Syphilis, by Dr. W. Lewin, of Friedrichsberg. From the German by P. W. Van Peyma, M. D., . . . . . 258

SELECTIONS:

Treatment of Puerperal Hemorrhage. By Dr. T. Gaillard Thomas, of New York, 261

EDITORIAL:

"By their Fruits ye Shall Know Them," . 272
The Erie County Medical Society—The Annual Meeting, . . . . . . 274
The Successor of Prof. James P. White. . 276
Editorial Notes, . . . . . . . . 276

REVIEWS, . . . . . . . 278

